# Effects of cadmium stress on growth and physiological characteristics of *sassafras* seedlings

**DOI:** 10.1038/s41598-021-89322-0

**Published:** 2021-05-10

**Authors:** Hongyi Zhao, Juelan Guan, Qing Liang, Xueyuan Zhang, Hongling Hu, Jian Zhang

**Affiliations:** 1 Institute of Ecology and Forestry, College of Forestry, Sichuan Agricultural University, Chengdu, 611130 China; 2 College of Forestry, Sichuan Agricultural University, Chengdu, 611130 China

**Keywords:** Ecology, Forestry, Environmental sciences

## Abstract

The effects of cadmium stress on the growth and physiological characteristics of *Sassafras tzumu* Hemsl*.* were studied in pot experiments. Five Cd levels were tested [CT(Control Treatment) : 0 mg/kg, Cd5: 5 mg/kg, Cd20: 20 mg/kg, Cd50: 50 mg/kg, and Cd100: 100 mg/kg]. The growth and physiological characteristics of the sassafras seedlings in each level were measured. The results showed that soil Cd had negative influences on sassafras growth and reduced the net growth of plant height and the biomass of leaf, branch and root. Significant reductions were recorded in root biomass by 18.18%(Cd5), 27.35%(Cd20), 27.57%(Cd50) and 28.95%(Cd100). The contents of hydrogen peroxide decreased first then increased while malondialdehyde showed the opposite trend with increasing cadmium concentration. Decreases were found in hydrogen peroxide contents by 10.96%(Cd5), 11.82%(Cd20) and 7.02%(Cd50); increases were found in malondialdehyde contents by 15.47%(Cd5), 16.07%(Cd20) and 7.85%(Cd50), indicating that cadmium stress had a certain effect on the peroxidation of the inner cell membranes in the seedlings that resulted in damage to the cell membrane structure. Superoxide dismutase activity decreased among treatments by 17.05%(Cd5), 10,68%(Cd20), 20.85%(Cd50) and 8.91%(Cd100), while peroxidase activity increased steadily with increasing cadmium concentration; these results suggest that peroxidase is likely the main protective enzyme involved in the reactive oxygen removal system in sassafras seedlings. Upward trends were observed in proline content by 90.76%(Cd5), 74.36%(Cd20), 99.73%(Cd50) and 126.01%(Cd100). The increase in proline content with increasing cadmium concentration indicated that cadmium stress induced proline synthesis to resist osmotic stress in the seedlings. Compared to that in CT, the soluble sugar content declined under the different treatments by 32.84%(Cd5), 5.85%(Cd20), 25.55%(Cd50) and 38.69%(Cd100). Increases were observed in the soluble protein content by 2.34%(Cd5), 21.36%(Cd20), 53.15%(Cd50) and 24.22%(Cd100). At different levels of cadmium stress, the chlorophyll content in the seedlings first increased and then decreased, and it was higher in the Cd5 and Cd20 treatments than that in the CT treatment. These results reflected that cadmium had photosynthesis-promoting effects at low concentrations and photosynthesis-suppressing effects at high concentrations. The photosynthetic gas exchange parameters and photosynthetic light-response parameters showed downward trends with increasing cadmium concentration compared with those in CT; these results reflected the negative effects of cadmium stress on photosynthesis in sassafras seedlings.

## Introduction

In recent years, environmental pollution and ecological damage have become increasingly serious due to the rapid development of industrialization. Among these issues, soil heavy metal pollution has become one of the most prominent environmental problems in the world. Cadmium (Cd) is a toxic, silver-white metal element. Because of its water solubility, fluidity, and toxicity, Cd can be easily absorbed by plant roots, and it can alter plant structural and functional properties, inhibit seed germination and root elongation^1^. Cd directly or indirectly inhibits physiological processes such as respiration, photosynthesis, water movement and gas exchange, leading to impairment in plant metabolism^2^. Additionally, Cd can affect the metabolism, chlorophyll synthesis^3^ of pants, and disturbs antioxidant defense system by increasing the production of reactive oxygen species (ROS)^4^.

The absorption and accumulation of Cd can affect the normal growth of plants. Cd stress leads to a decline in biomass and photosynthetic rate of plants, also causes oxidative damage and imbalance of nutrient uptake^5,6^. Apart from inhibiting the formation of photosynthetic pigments, reducing the efficiency of photosynthesis, Cd stress can also increase the accumulation of active oxygen and enhance peroxidation^7,8^. Plants can reduce the stress caused by heavy metals through the generation and synthesis of various enzymatic antioxidants, non-enzymatic antioxidants, osmolytes and chelating agents^9^. Because that Cd can accumulate in plants and enter human body through the food chain, causing chronic poisoning and endangering human health^10^, it is of great importance to find an effective method to remediate Cd-contaminated soils.

Woody plants have proved an effective means for removing or stabilizing toxic metals from contaminated soils, with their high accumulation of heavy metals, perennial trait, high biomass production, and fast growth^6,11–13^. *Sassafras tzumu* Hemsl. belongs to the genus *Sassafras* and the family Lauraceae. It is often used for shipbuilding and making high-quality furniture. Sassafras trees are beautiful and have red leaves in autumn, which makes them excellent ornamental trees for gardens^14,15^. Sassafras has good prospects in terms of timber production and garden use. Therefore, most studies on sassafras in China have focused on genetic breeding and reproduction technology, cultivation and afforestation technology, disease and pest control, etc.^16–18^. However, few studies have been performed on the physiological conditions of sassafras under heavy metal stress. In view of this, this study aimed to investigate the effects of Cd stress on the growth, photosynthetic and physiological characteristics of sassafras and to analyze the physiological response of sassafras to Cd stress in order to provide a theoretical basis for further research on the resistance of sassafras to Cd stress.

## Materials and methods

### Experimental design

The plants used in this experiment were 1-year sassafras seedlings, which are common native tree species in Sichuan. The seedlings were obtained from the same source and were pests and diseases free, which were collected in accordance with relevant national and international guidelines and legislations. The tested soil was a yellow soil taken from a forest farm in Ya’an city, Sichuan Province. The pH of the test soil was 5.75, and the total nitrogen, phosphorus and potassium contents were 9.10 g/kg, 0.64 g/kg, and 12.59 g/kg, respectively.

The experiment was conducted at the teaching and research station of Sichuan Agricultural University. Its geographical coordinates are east longitude 103°51′29″ and north latitude 30°42′18″. Approximately 12 kg of mixed soil was weighed and placed into each pot. The content of Cd^2+^ (mg/kg) in the soil of each pot was calculated according to the dry weight of the soil, and Cd^2+^ was added to the pots in the form of a CdCl_2_ aqueous solution. A single-factor test design was used to establish 5 cadmium treatment levels: CT (0 mg/kg), Cd5 (5 mg/kg), Cd20 (20 mg/kg), Cd50 (50 mg/kg), and Cd100 (100 mg/kg). Five replicates were established for each treatment group, and all treated plants were placed in a greenhouse.

The experiment began in early May 2019. CdCl_2_ was applied 5 times, with an interval of 15 d; the same amount of pure water was added to CT. At the time of application, the prepared CdCl_2_ solution was poured evenly onto the soil surface in the basin. Any CdCl_2_ solution that exuded from the pot was collected at the bottom of the basin with a tray pad and poured back into the soil. The CdCl_2_ applications ended in mid-July 2019. After 30 d of plant growth, the plant photosynthetic and physiological characteristics were measured. The plants were harvested at the end of December 2019, and the plant roots, branches and leaves were harvested separately.

### Determination of the plant growth index

The height and ground diameter of each plant were measured before and after the experiment, and the subtraction method was used to calculate the results. The height of the seedlings was measured with a ruler (precision: 0.1 cm). An electronic Vernier caliper (precision: 0.1 mm) was used to measure the ground diameter from two perpendicular directions at the root neck, and the average value was calculated. The plant samples were washed with deionized water, and then the plant organs, i.e., roots, branches and leaves, were harvested separately. In the lab, the plant organs were put in an oven at 105 °C for 30 min, after which they were dried at 70 °C to constant weight and weighed. The biomass of each dried organ was then calculated.

### Determination of the physiological characteristics

In the 5 replicates of each treatment, five mature fresh functional leaves were randomly selected from the middle to upper part of the tree canopy and placed separately into an ice box for the measurement of each index.

The content of free proline (Pro) was extracted with sulfonyl salicylic acid and determined with acidic ninhydrin colorimetry^19^. The content of soluble protein (SP) was determined by the Coomassie brilliant blue method^20^. The malondialdehyde (MDA) and soluble sugar contents (SS) were determined by the thiobarbituric acid heating colorimetric method^20,21^.

The activity of superoxide dismutase (SOD), Peroxidase (POD) and catalase (CAT) were determined by the spectrophotometer. 0.2 g of the fresh leaves were weighed and placed in a grinding bowl, 2 ml phosphate buffer solution was added and placed in an ice bath for rapid grinding, then centrifuge at 10,000 r/min at 4 °C for 15 min, the supernatant was collected as the test sample. According to the method of Donahue et al.^22^, the OD value at the wavelength of 560 nm causes 50% inhibition of the reduction rate of nitro blue tetrazolium (NBT) to measure the SOD activity. POD activity was measured by the Guaiacol oxidation method^23^, and the increase in absorbance at a wavelength of 470 nm was determined. CAT activity was quantified as the decrease in the absorbance at a wavelength of 240 nm caused by the consumption of the substrate H_2_O_2_^24^. The H_2_O_2_ content was determined with a method based on H_2_O_2_ and titanium ions forming a colored [TiO(H_2_O_2_)]^2+^ coordination compound (the specific absorption peak is 410 nm). All enzyme activities were determined with kits produced by Nanjing Jiancheng Biological Research Company.

### Determination of the chlorophyll content

Two to three mature functional leaves of the plants in each treatment were randomly selected for the determination of chlorophyll content. During the determination, after removing the veins of the leaf, cut the remaining parts into pieces, and store the samples in refrigerator for preservation. An amount of 0.1 g leaves was accurately weighed and placed into a 10 mL centrifuge tube. An amount of 9 ml chlorophyll extraction solution (80% acetone and anhydrous ethanol 1:1 mix) was added, and the mixture was placed in the dark and left for more than 24 h until the leaves were completely white^26^. Spectral measurements were performed at wavelengths of 663 nm and 646 nm.

### Determination of the photosynthetic parameters

The net photosynthetic rate (*Pn*), stomatal conductance (*Gs*), transpiration rate (*Tr*), and intracellular CO_2_ concentration (*Ci*) were determined with an LI-6800 portable photosynthesis system (Li-Cor Inc, USA). Three plants were selected from each treatment, three leaves were selected from each plant, and ten data points were recorded for each leaf.

### Determination of the photosynthetic light-response curve

The LI-6800 portable photosynthesis system (Li-Cor Inc, USA) was used to measure the photosynthetic light-response curves of the leaves selected for the previous determination. The photosynthetically active radiation (PAR) gradient values were 1800, 1600, 1000, 800, 600, 400, 200, 100, 75, 50, 25, and 0 μmol/m^2^/s, the CO_2_ concentration was set to 400 ppm, and the room temperature was set to 30 °C. Each leaf was photoinduced for 20 min before the determination, and in this test, the photoinduction strength was 800 μmol/m^2^/s.

The photosynthetic light-response curves of sassafras leaves treated with different concentrations of cadmium were fitted by using Ye’s^27^ modified linear hyperbolic model. The fitting equation of the modified linear hyperbolic model is as follows:

$$ Pn = \alpha \frac{1 - \beta I}{{1 - \gamma I}}I - Rd $$

When *Pn* = 0, the light compensation point (*LCP*) can be obtained:

$$ LCP = \frac{{\alpha - Rd\gamma - \sqrt {\left( {\alpha - Rd} \right)^{2} - 4\alpha \beta Rd} }}{2\alpha \beta } $$

With d*Pn*/dI = 0, the light saturation point (*LSP*) can be obtained:

$$ LSP = \frac{{\sqrt {\left( {\beta + \gamma } \right)/\beta } - 1}}{\gamma } $$

When I = Is, *Pn*_*max*_ can be obtained:

$$ Pn\max = \alpha \frac{1 - \beta Is}{{1 + \gamma Is}}Is - Rd $$

where *Pn* is the net photosynthetic rate; *Pn*_*max*_ is the maximum net photosynthetic rate at light saturation; α is the initial quantum efficiency; I is the photosynthetically active radiation; Rd is the dark respiration rate; β is the light suppression coefficient, which is the correction coefficient; and γ is the light saturation coefficient, which is the ratio of the initial slope of the light-response curve of the plant leaves to the maximum net photosynthetic rate.

### Statistical analysis

All data were collected in Excel 2016 and statistically analyzed with SPSS 24.0. The significance of the differences among means was assessed by one-way analysis of variance (ANOVA). Multiple comparisons were performed mostly with Duncan’s test to compare the mean values between treatments (*p* < 0.05). The figures were drawn in Origin 8.5.

## Results

### The effects of Cd stress on plant growth

The net growth in plant height decreased with increasing Cd concentration, but there were no significant differences among treatments (*P* < 0.05). The net growth in ground diameter showed an increasing trend, but there were no significant differences among treatments (*P* < 0.05). Significant reductions were recorded in root biomass by 18.18%, 27.35%, 27.57% and 28.96%, respectively among treatments compared to CT. The biomass of leaves and branches decreased with the aggravation of cadmium stress, and there were significant differences between treatments and CT for branches (except in Cd5); however, no significant differences were found for leaf biomass (*P* < 0.05). Declines were found in total biomass by 11.01%, 18.92%, 21.14% and 27.72%, respectively in sassafras under different Cd treatments. (Table 1).

**Table 1:** The growth characteristics of sassafras seedlings under different levels of cadmium treatment.

Treat-ment	Net growth in plant height/cm	Net growth in ground diameter/cm	Leaf biomass/g	Branch biomass/g	Root biomass/g	Total biomass/g
CT	36.19 ± 3.58a	4.12 ± 0.83a	25.94 ± 0.77a	45.46 ± 1.23a	48.45 ± 1.15a	119.85 ± 2.73a
Cd5	24.89 ± 0.51a	3.38 ± 0.92a	22.79 ± 1.50a	44.22 ± 0.96a	39.64 ± 1.25b	106.65 ± 1.42ab
Cd20	33.03 ± 6.49a	7.11 ± 1.73a	21.01 ± 9.20a	40.96 ± 1.28b	35.20 ± 2.80b	97.17 ± 7.76ab
Cd50	29.2 ± 3.15a	6.58 ± 1.17a	20.58 ± 2.03a	38.84 ± 0.09b	35.09 ± 1.44b	94.51 ± 3.26bc
Cd100	23.65 ± 3.13a	6.71 ± 1.04a	19.39 ± 1.54a	32.82 ± 1.00c	34.42 ± 1.44b	86.63 ± 1.32c

Different lowercase letters in the same column indicate significant differences between different cadmium concentration treatments (*P* < 0.05).

### The effect of Cd stress on physiological characteristics

#### The contents of H_2_O_2_ and MDA in sassafras leaves under Cd stress

**Figure 1:**
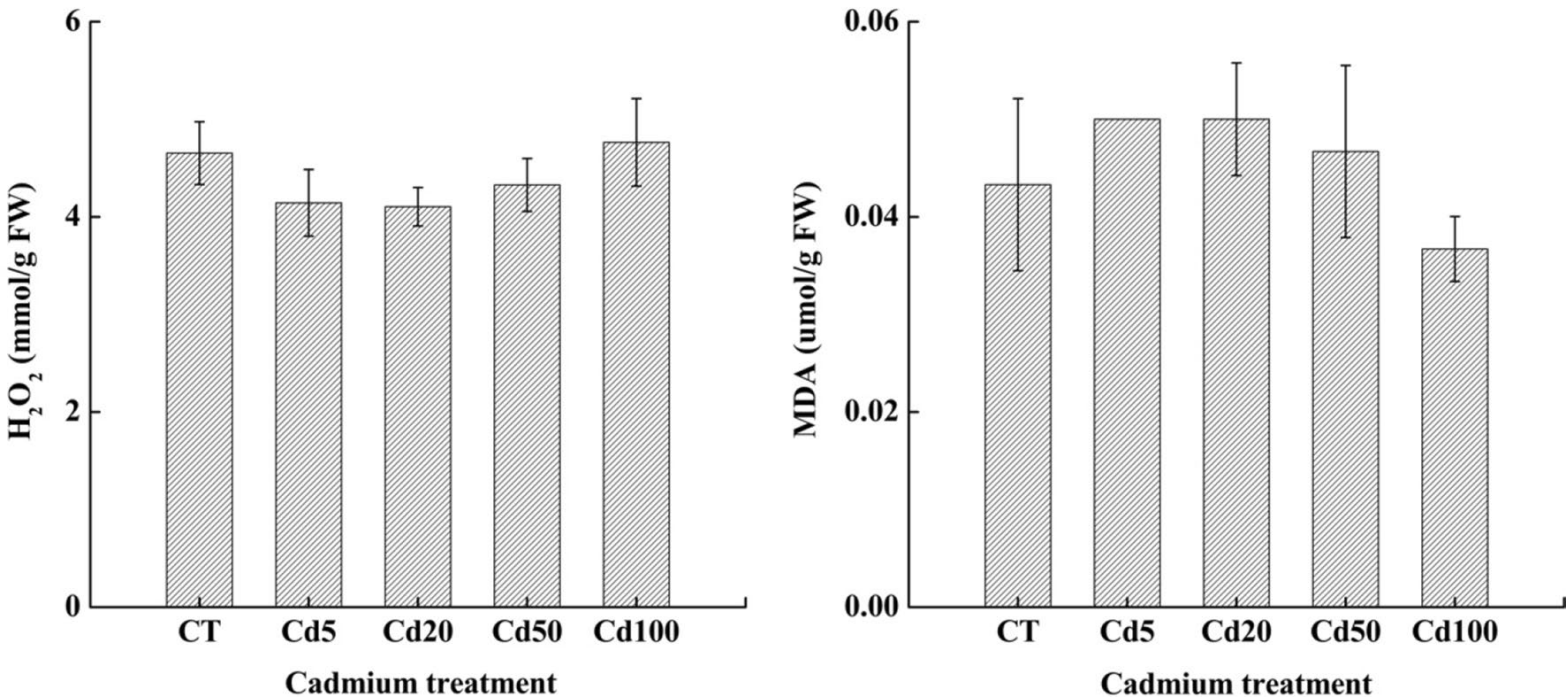
Changes in the hydrogen peroxide (H_2_O_2_) and malondialdehyde (MDA) contents of sassafras leaves under different levels of cadmium treatment. Different lowercase letters over the columns indicate significant differences between treatments (*P* < 0.05), no lowercase letters indicate there’s no significant differences between treatments, the same below.

The H_2_O_2_ content decreased first by 10.96%, 11.82% and 7.02% respectively in Cd 5, Cd 20 and Cd50, and then increased by 2.36% in Cd100, but there were no significant differences among treatments. The MDA content showed the opposite trend to the H_2_O_2_ content as the cadmium concentration increased; increases were found in Cd 5, Cd 20 and Cd50 by 15.47%, 16.07% and 7.85%, respectively, and declined by 15.24% in Cd100; however, there were still no significant differences among treatments. (Fig. 1).

#### Response of antioxidant enzyme activity to Cd stress

**Figure 2:**
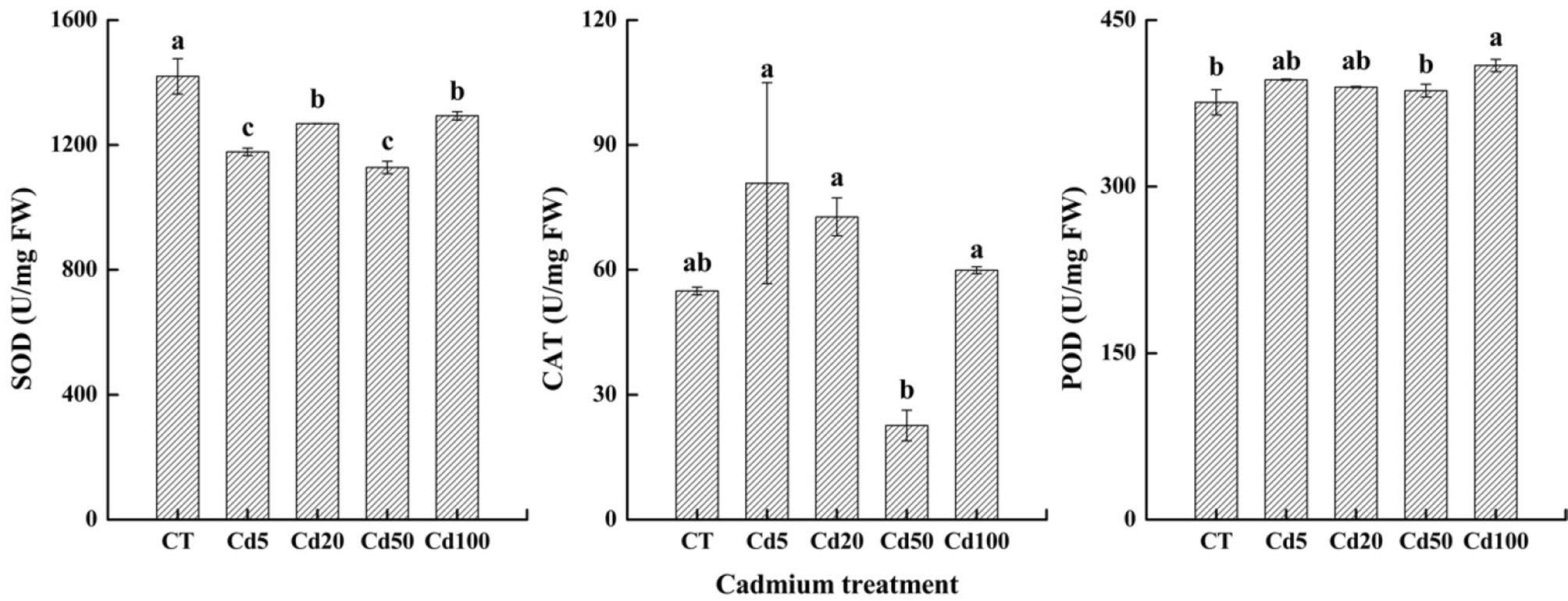
Changes in superoxide dismutase (SOD), catalase (CAT) and peroxidase (POD) activities in sassafras leaves under different levels of cadmium treatment.

SOD activity fluctuated with the increasing cadmium concentration, while the CT treatment showed the highest SOD activity, decreases were found by 17.05%, 10.68%, 20.85% and 8.91%, respectively among treatments. CAT activity showed a fluctuation trend, there were increases found in Cd5, Cd20 and Cd 100 by 47.19%, 32.45% and 9.06%, and decreased by 58.77% in Cd50. POD activity showed a slowly increasing trend, and the highest activity was observed in the Cd100 treatment. The increases of POD activity were recorded in sassafras by 5.41%, 3.66%, 2.81% and 8.83%, respectively in Cd5, Cd20, Cd50 and Cd100. (Fig. 2).

#### Changes in the contents of osmotic regulation substances in response to Cd stress

**Figure 3:**
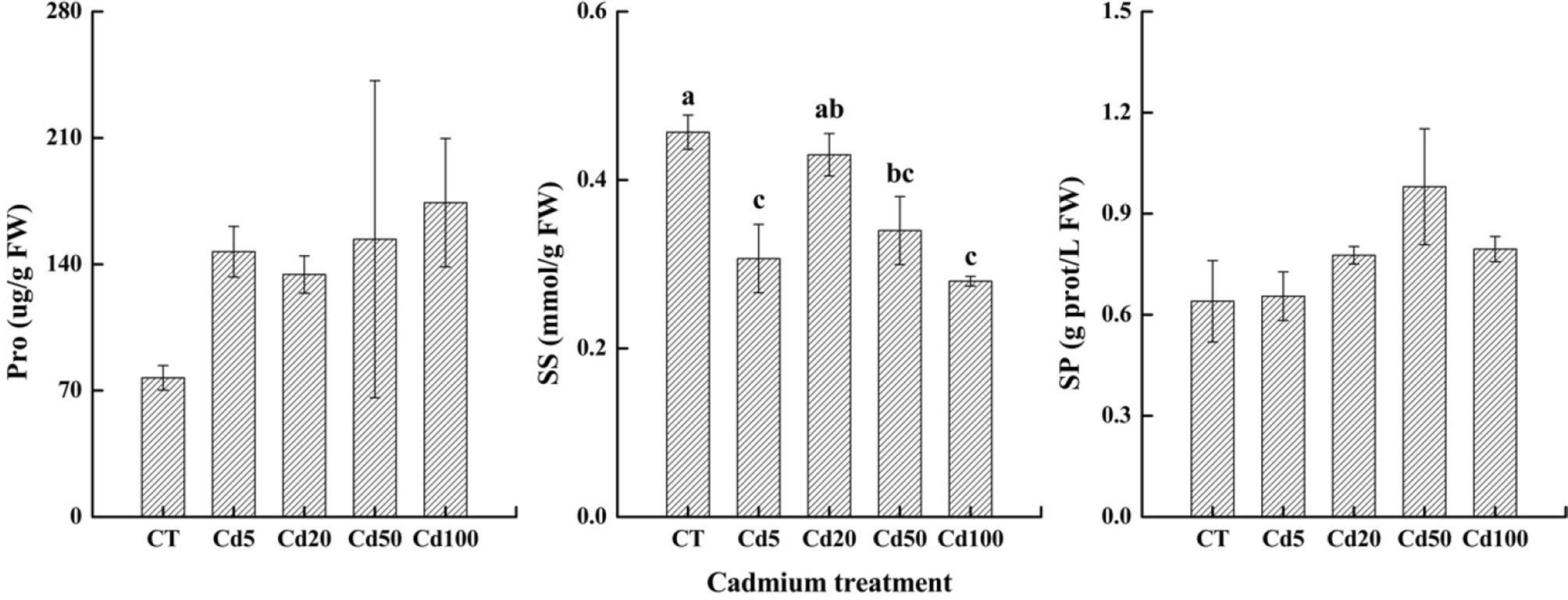
Changes in proline (Pro), soluble sugar (SS) and soluble protein contents (SP) in sassafras leaves under different levels of cadmium treatment.

Upward trends were observed in Pro content by 90.76%, 74.36%, 99.73% and 126.01%, respectively among treatments; the Cd100 treatment had the maximum Pro content, but there were no significant differences among treatments. The content of SS was the highest in the CT treatment, while the SS content in the other treatments decreased compared to CT. Decreases were found in SS content by 32.84%, 5.85%, 25.55% and 38.69%, respectively. The SP content first increased and then decreased with increasing cadmium concentration and reached a maximum in the Cd50 treatment. Compared to CT, there were increases recorded in SP content by 2.34%, 21.36%, 53.13% and 24.22%, respectively; however, the differences between treatments were not significant. (Fig. 3).

### The effects of Cd stress on photosynthetic characteristics

#### Chlorophyll content in sassafras leaves under cadmium stress

**Figure 4:**
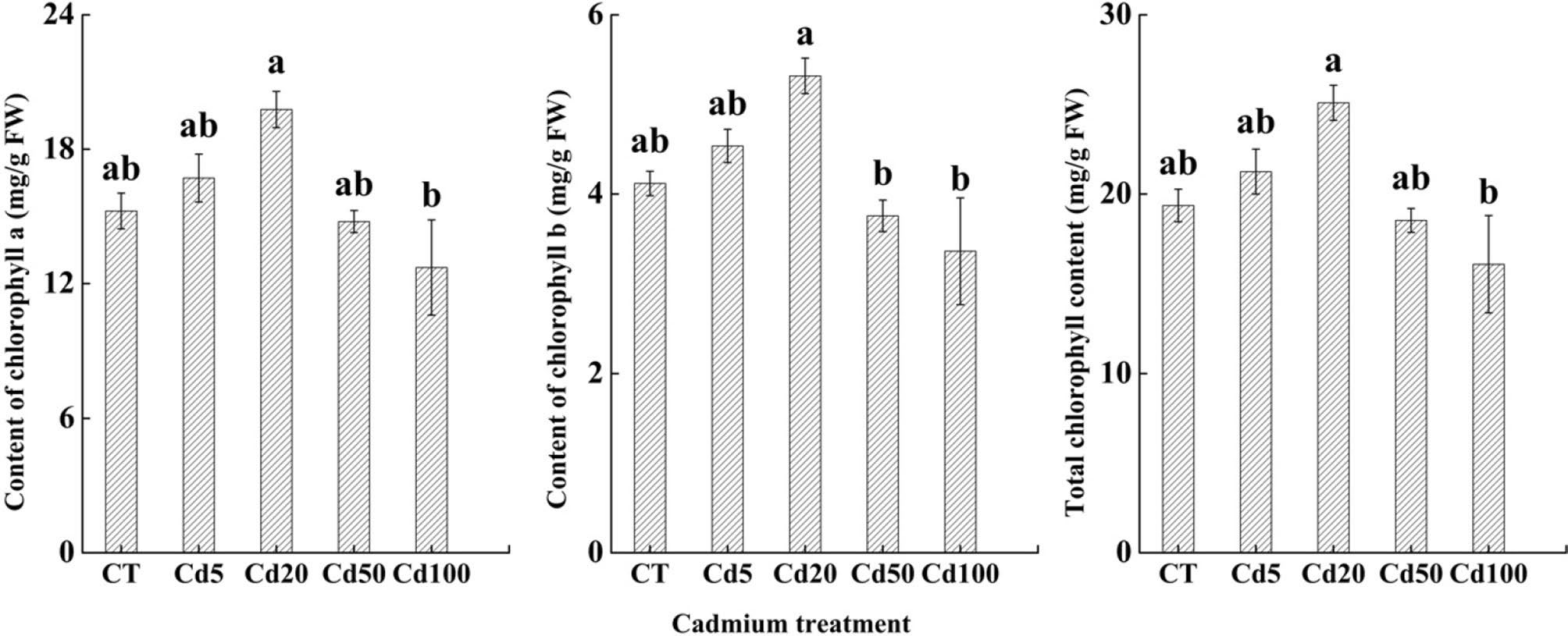
Chlorophyll content of sassafras leaves under different concentrations of cadmium.

With the increase in the cadmium concentration, the chlorophyll a and b contents and the total chlorophyll content in sassafras leaves first increased and then decreased, reaching a maximum in Cd20. There were no significant differences in the chlorophyll a content or the total chlorophyll content under the Cd5, Cd20 and Cd50 treatments, while the Cd100 treatment was significantly different from CT (*P* < 0.05). The chlorophyll b content showed a similar change trend as the chlorophyll a content, but a significant difference between the chlorophyll a and b contents appeared in Cd50 (*P* < 0.05). (Fig. 4).

#### Changes in photosynthetic gas exchange parameters in sassafras under cadmium stress

**Figure 5:**
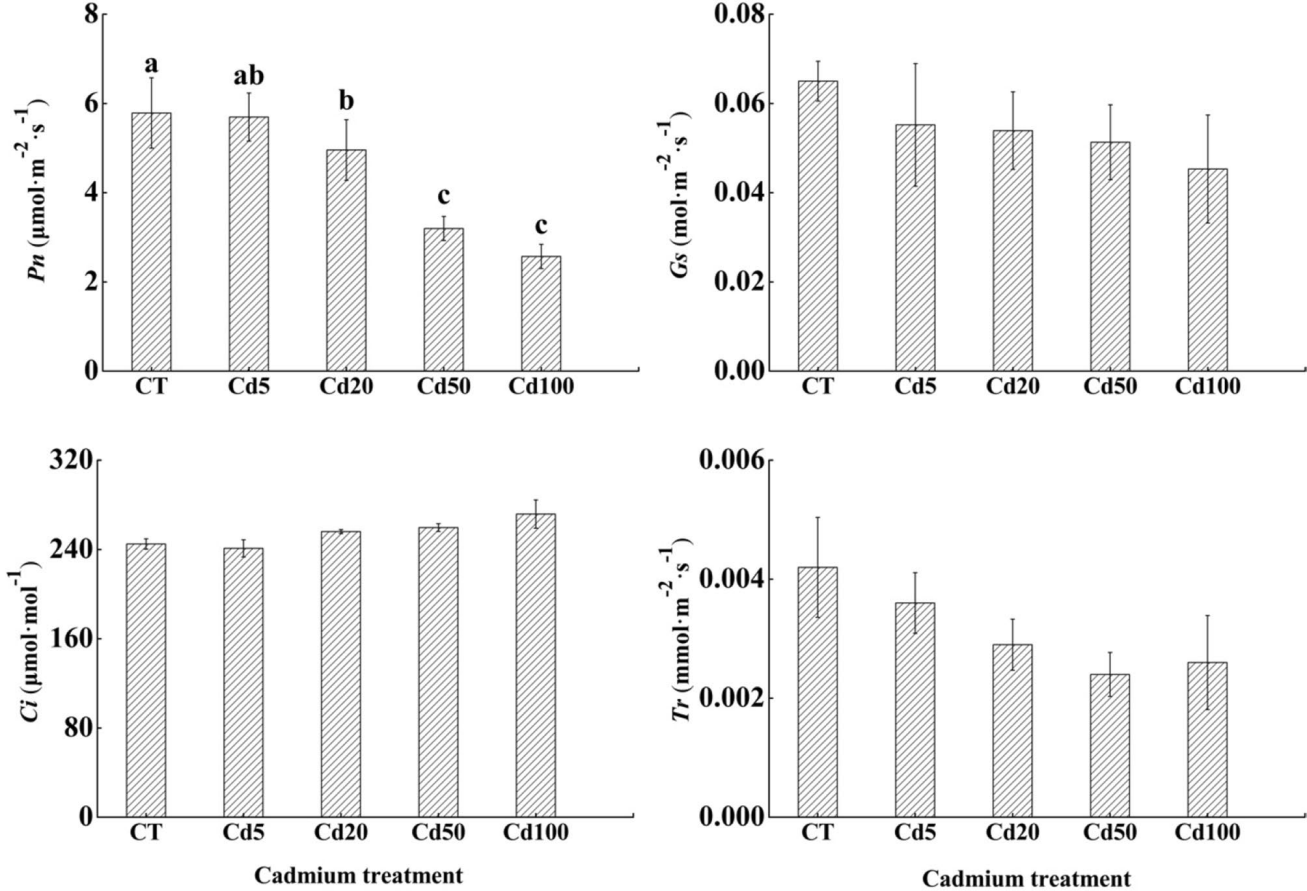
Photosynthetic gas exchange parameters of sassafras leaves under different cadmium stress treatments.

With the increase in Cd concentration, the *Pn* in the sassafras’ leaves showed a downward trend. Significant reductions were found in the *Pn* of sassafras’ leaves by 1.60%, 14.35%, 44.74% and 55.57%, respectively among treatments. The different concentrations of Cd did not significantly affect *Gs*, *Ci* or *Tr*. There were decreases found in *Gs* by 15.08%, 17.08%, 21.08% and 30.31%, respectively. Declines were also recorded in *Tr* by 14.29%, 30.95%, 42.86% and 38.10%, respectively. (Fig. 5).

#### The effect of Cd stress on the photosynthetic light-response curve

**Figure 6:**
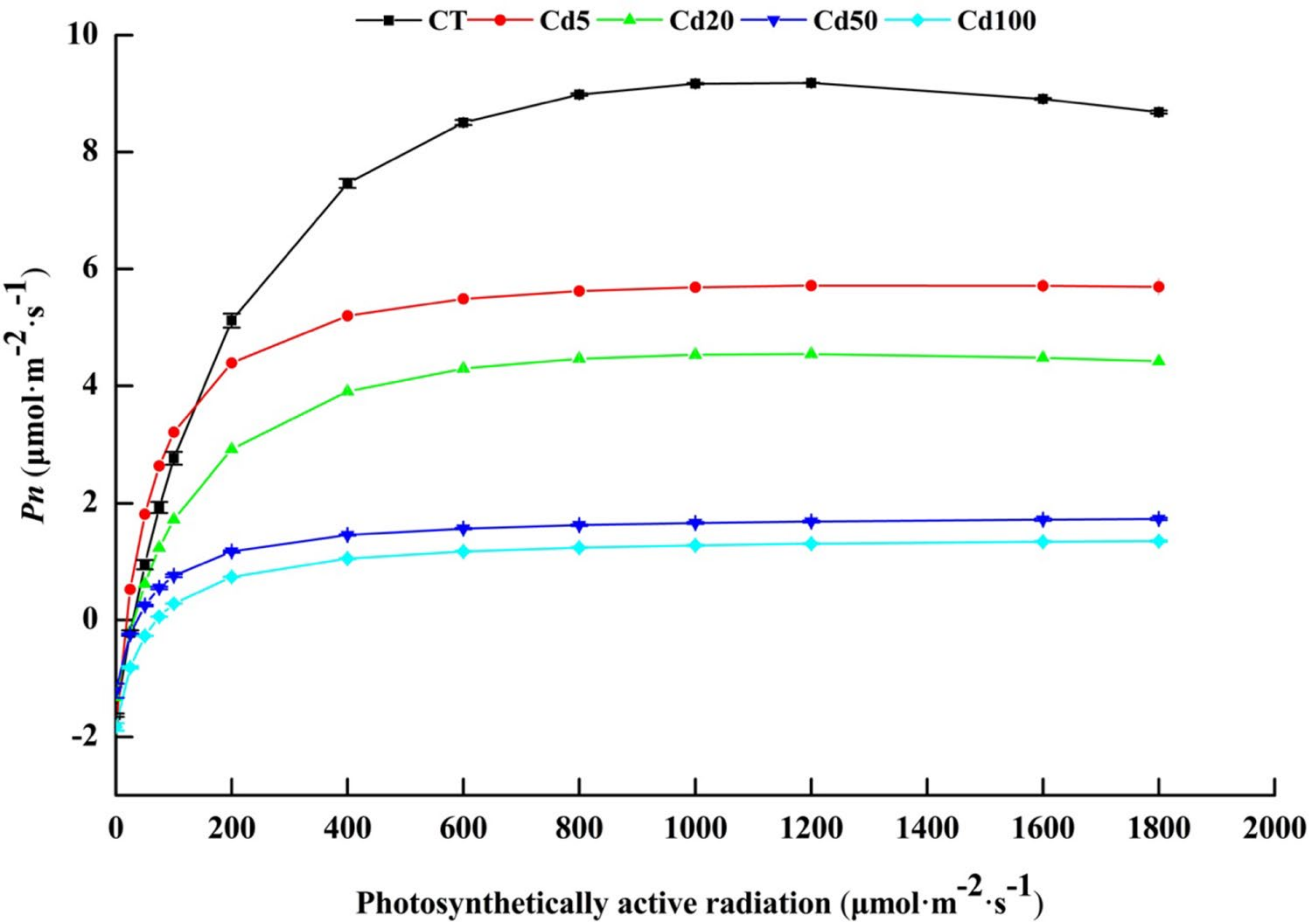
Photosynthetic light-response curves of sassafras leaves under the different cadmium stress treatments.

When the PAR was weak (*PAR* ≤ 200 μmol/m^2^/s), the *Pn* of leaves showed a nearly linear increasing trend, the increase in the *Pn* in CT was significantly higher than that in the cadmium treatments, and the increase in the *Pn* of Cd5, Cd20, Cd50 and Cd100 decreased with the increase in cadmium concentration. The increasing trend of the *Pn* of leaves slowed under different Cd concentrations when the PAR exceeded 200 μmol/m^2^/s. When the PAR was between 800 ~ 1800 μmol/m^2^/s, the *Pn* of leaves tended to be stable and eventually reached the light saturation point when leaf photosynthesis exhibited photoinhibition (Fig. 6).

The modified orthogonal hyperbolic model from Ye^27^ was used to fit the photosynthetic response curves of the sassafras leaves at the different cadmium concentrations, and the characteristic parameters of the light response were calculated (Table 2).

**Table 2:** Photosynthetic light-response parameters.

Treatment	*Pn*_max_ (µmol/m^2^/s)	α	*Rd* (µmol/m^2^/s)	*LCP* (µmol/m^2^/s)	*LSP* (µmol/m^2^/s)
CT	9.19 ± 0.007a	0.06 ± 0.003b	1.63 ± 0.029abc	29.47 ± 1.015a	1113.86 ± 11.919a
Cd5	5.78 ± 0.139b	0.13 ± 0.006a	1.81 ± 0.112ab	17.92 ± 0.968a	2326.13 ± 1157.22a
Cd20	4.56 ± 0.043c	0.05 ± 0.003b	1.32 ± 0.161bc	30.37 ± 3.252a	1182.54 ± 100.105a
Cd50	1.82 ± 0.027d	0.06 ± 0.008b	1.21 ± 0.122c	105.59 ± 70.914a	868.16 ± 3.473a
Cd100	1.45 ± 0.014e	0.06 ± 0.003b	1.83 ± 0.065a	− 42.29 ± 112.027a	723.38 ± 2.234a

*Pn*_*max*_: Maximum net photosynthetic rate; α: Initial quantum efficiency; *Rd*: Dark respiration rate; *LCP*: Light compensation point; *LSP*: Light saturation point. R^2^: 0.94–0.99. Different lowercase letters in the same column indicate significant differences between treatments with different cadmium concentrations.

The maximum net photosynthetic rate (*Pn*_max_) of the leaves decreased significantly with increasing Cd concentration. The highest *Pn*_max_ appeared in CT, at 9.19 μmol/m^2^/s; then, *Pn*_max_ decreased in the different treatments, by 37.11% (Cd5), 50.38% (Cd20), 80.20% (Cd50) and 84.22% (Cd100). The dark respiration rates (*Rd*) of the sassafras leaves under the different Cd concentrations were not significantly different from that of CT (Table 2).

The light compensation point (*LCP*) of the leaves was the highest in the Cd50 treatment and the lowest in the Cd100 treatment; however, there were no significant differences among the treatments. The light saturation point (*LSP*) of the leaves was the highest at Cd5 and the lowest at Cd100, and there were also no significant differences among the treatments (Table 2).

## Discussion

Cadmium (Cd) is highly mobile in plant tissues and affects plant physiological growth^28^. This study demonstrated that Cd stress in soil had a negative influence on sassafras growth and reduced the net growth in plant height and leaf, branch and root biomass. The decrease of the net growth in plant height and biomass may have been due to the toxicity of Cd to photosynthetic organs^29^ and plant structure^30^. Under Cd stress, the biomass allocation pattern of sassafras also changed; the degree of the decline in aboveground biomass was higher than that of the decline in underground biomass, probably because cadmium stress limited the acquisition of mineral nutrients required for plants^31^. These effects intensified the competition for nutrients among different plant organs; therefore, the plants retained their underground biomass at the expense of aboveground biomass in order to obtain more soil nutrients and maintain normal growth. In addition, cadmium stress can limit and inhibit plant photosynthesis, which weakens their photosynthetic production capacity and ultimately reduces their biomass; these impacts were more evident on aboveground biomass than on underground biomass^32^.

Cd toxicity is known to cause a deleterious effect on plants by disturbing the overall physiological mechanisms of plants^33^. It is found that heavy metals can stimulate plants to produce more reactive oxygen species (ROS), the produced ROS react with lipids, proteins, nucleic acids and other substances, causing lipid peroxidation, membrane damage and enzyme inactivation, thereby affecting cell performance and viability^34^. Once the ability of the plant to clear itself is exceeded, it will also cause the plant to die. When plants are subjected to abiotic stress, H_2_O_2_, the most abundant and stable type of ROS, plays a key regulatory role in their organs and protects the plants from the harm caused by the abiotic stress^35,36^. The MDA content, which is an indicator of lipid peroxidation in cellular organelles, is usually upregulated in response to various external stimuli^37^. In this study, the H_2_O_2_ and MDA contents showed upward trends, there were no significant differences among the treatments. It might because that cadmium stress had a certain effect on the peroxidation of the inner membrane of sassafras seedlings, resulting in damage to the cell membrane structure. Some studies have noted that an increase in the activity of the protective enzyme system reduces lipid membrane peroxidation and maintains the integrity of the membrane system^38^. The primary antioxidant enzymes are SOD, CAT and POD. When plants are subjected to heavy metal stress, the activity of antioxidant enzymes increases gradually with increasing heavy metal concentration, but when the heavy metal concentration becomes too high, the protective enzyme system is destroyed, and enzyme activity decreases^39^. The resistance of antioxidant enzymes to heavy metal stress is a complex physiological process that is influenced by the plant species and by the concentration and properties of the heavy metal. In Ozfidan-Konakci C’s research, the POD content in wheat leaves increased significantly under cadmium stress, but the SOD content did not change^40^. In this study, SOD activity decreased among treatments compared to CT, while POD activity increased steadily with increasing cadmium concentration. These results indicated that POD was likely the main protective enzyme involved in the reactive oxygen removal system in sassafras. The results also indicate that sassafras adapts to the increase in reactive oxygen species (ROS) and enhances its tolerance to cadmium by adjusting the activities of SOD and POD in its organs under cadmium stress, eliminating harmful substances such as O_2_^-^ and H_2_O_2_ to maintain the normal metabolism of free radicals in plants^41^. At the same time, the increased toxicity of Cd could inhibit the reaction ability of the antioxidant system^42^. However, when the cadmium content exceeded a certain range, the activities of SOD, CAT and POD in the stressed plants were inhibited, resulting in a limited ability to remove ROS and serious damage to the functional membranes and enzyme systems of the plant tissues and cells.

Pro is an important osmotic protective substance that plays an indispensable role in maintaining the normal function of cells, protecting the structure of cell membranes, and the stability of biological macromolecule structures^43^. Heavy metal stress impacts the water balance in plants and induces a large increase in Pro, which is involved in the osmotic regulation of cells^44^. Some studies have shown that a variety of abiotic stresses tend to make plants produce a large amount of Pro and accumulate Pro in their organs^45,46^. Plants have different pathways for the synthesis and degradation of Pro in different situations, so the effects of Pro are not completely consistent^47,48^. In this study, the content of Pro increased with increasing cadmium concentration, indicating that cadmium stress induced sassafras to synthesize more Pro to resist osmotic stress. SS is not only the product of plant photosynthesis but also participates in the process of plant photosynthesis, which can provide energy for the growth and development of plant organs and plays an important role in plants. SS can reduce the osmotic potential in cells and maintain cell water potential and normal metabolism^49,50^. In this study, compared with that in CT, the SS content declined under different concentrations of cadmium. This result may have been due to the destruction of chloroplasts and the subsequent decrease in photosynthesis with the aggravation of the degree of stress. At the same time, to resist the toxicity of heavy metals, plant tissue cells enhance their metabolic activities and consume some of the SS. Cd stress can affect the synthesis of normal proteins and the production of stress proteins. SP can increase the amount of functional protein to maintain normal physiological metabolic activities of cells, leading to the improvement of plants resistance to stress^51^. According to this research, as the concentration of cadmium increased, the SP content first increased and then decreased, which was similar to the research results of Ge Wei et al.^52^. It may be that when the cadmium content is low, mild cadmium stress causes young trees to produce more antioxidant proteins and stress proteins in response to cadmium toxicity. However, with the aggravation of cadmium stress, the protein synthesis system is damaged to a certain extent. In addition, cadmium stress inhibits plant photosynthesis, resulting in a decrease in the contents of proteins involved in ATP activities; therefore, the content of SP decreases^53,54^.

Photosynthesis is an important physiological process in plants. Many studies have shown that cadmium stress can inhibit plant photosynthesis and is significantly related to the degree of heavy metal stress^55,56^. Its effects on plant photosynthesis are mainly reflected in the destruction of chlorophyll structure and the reduction in photosynthetic pigment content, which can affect the ability of plants to photosynthesize^57^. The chlorophyll content is directly related to the intensity of photosynthesis and can reflect the ability of leaves to absorb and transform light energy. As the main pigment involved in photosynthesis, chlorophyll a is responsible for converting light energy into chemical energy in the light reaction center, while chlorophyll b is responsible for capturing and transmitting light energy^58^. In this research, the chlorophyll content first increased and then declined at increasing cadmium concentrations, which indicated that a low concentration of cadmium promotes an increase in chlorophyll content, while a high concentration of cadmium inhibits the formation of chlorophyll. This was probably due to the low concentration of Cd complexes accelerating the absorption of Mg, Fe, K and P nutrients from the soil, promoting the formation of leaf porphyrin rings, and thus increasing the chlorophyll content^59^.

Studies have shown that the *Pn* of plants generally decreases with increasing heavy metal concentration^60^, which is in line with our experimental results. Factors that affect the photosynthetic rate of plant leaves can be divided into stomatal factors and nonstomatal factors. Farquhar et al. proposed that to judge the factors that affect the photosynthetic rate of plants, changes in stomatal conductance and intercellular carbon dioxide concentration should be observed at the same time^61^. In this experiment, the *Pn* and *Gs* of sassafras leaves decreased, while the *Ci* increased, indicating that the photosynthetic rate of sassafras leaves was limited by nonstomatal factors.

Photosynthetic light-response parameters can reflect the photosynthetic potential, light energy utilization and light inhibition of plants under adverse conditions^27,62^. *Pn*_max_ can reflect the potential photosynthetic capacity of plants. The greater the value of *Pn*_*max*_, the greater the photosynthetic potential of plant leaves, indicating that leaves can synthesize more photosynthetic products under the appropriate light conditions^63^. In this study, the *Pn*_max_ of sassafras leaves decreased significantly with increasing Cd stress, indicating that heavy metal stress significantly affected the photosynthetic capacity of the sassafras leaves. The *LCP* and *LSP* reflect the ability of plants to utilize weak light and strong light, respectively^64^. In this study, with the increase in Cd concentration, the *LCP* and *LSP* of sassafras leaves generally showed a decreasing trend, indicating that Cd stress weakened the ability of sassafras leaves to use both weak light and strong light. Qi’s research showed that reducing the *LCP* was an adaptive response of plants to a low light environment and was conducive to maintaining the carbon balance of plants under low light intensity^65^. Lower *Rd* and *LCP* in leaves are more conducive to reducing the consumption of photosynthetic products in leaves, thus increasing the net photosynthetic accumulation to obtain the maximum carbon accumulation^63^. Under cadmium stress, sassafras leaves maintained normal photosynthesis by reducing the *Rd* and *LCP*, which reflected the mechanism of the response of sassafras to cadmium stress.

## Conclusions

Cd stress had negative influences on sassafras growth, as it reduced the net growth of plant height and the biomass of leaf, branch, and root. The contents of H_2_O_2_ decreased first then increased while MDA showed the opposite trend with increasing cadmium concentration, which indicated that cadmium stress had a certain effect on the peroxidation of the inner membrane of sassafras seedlings, resulting in damage to the cell membrane structure. SOD activity declined among treatment compare to CT, while POD activity increased steadily with increasing cadmium concentration, indicating that POD was likely the main protective enzyme involved in the reactive oxygen removal system in sassafras. The increase in Pro content indicated that cadmium stress induced sassafras to synthesize more Pro to resist osmotic stress. Compared to that in CT, the SS content declined under the different Cd treatments. The SP content increased with the increasing levels of cadmium stress. At increasing levels of cadmium stress, the chlorophyll content of the seedlings first increased and then decreased, and it was higher in the Cd5 and Cd20 treatments than in the CT treatment. These results reflected that Cd had photosynthesis-promoting effects at low Cd concentrations and photosynthesis-suppressing effects at high Cd concentrations. The photosynthetic gas exchange parameters and photosynthetic light-response parameters showed downward trends in the Cd treatments compared with those in CT, which reflected the inhibition of photosynthesis in sassafras due to Cd stress.

## Abbreviations

Cd: Cadmium
Chl a: Chlorophyll a
Chl b: Chlorophyll b
Car: Carotenoids
Chl: Chlorophyll
PAR: Photosynthetically active radiation
*P*_*n*_: Net photosynthetic rate
G*s*: Stomatal conductance
*T*_r_: Transpiration rate
*C*_*i*_: Intercellular CO_2_ concentration
*LSP*: Light saturation point
*Rd*: Dark respiration rate
*LCP*: Light compensation point
SOD: Superoxide dismutase
POD: Peroxidase
Pro: Proline
SS: Soluble sugar
SP: Soluble protein
CAT: Catalase
MDA: Malondialdehyde
ROS: Reactive oxygen species
